# Leukocyte Indices as Markers of Inflammation and Predictors of Outcome in Heart Failure with Preserved Ejection Fraction

**DOI:** 10.3390/jcm13195875

**Published:** 2024-10-02

**Authors:** Michael Poledniczek, Christina Kronberger, Luca List, Bernhard Gregshammer, Robin Willixhofer, Nikita Ermolaev, Franz Duca, Christina Binder, René Rettl, Roza Badr Eslam, Luciana Camuz Ligios, Christian Nitsche, Christian Hengstenberg, Johannes Kastner, Jutta Bergler-Klein, Andreas Anselm Kammerlander

**Affiliations:** Division of Cardiology, Department of Internal Medicine II, Medical University of Vienna, 1090 Vienna, Austria; michael.poledniczek@meduniwien.ac.at (M.P.);

**Keywords:** heart failure with preserved ejection fraction, systemic inflammation, leukocyte indices

## Abstract

**Background:** The pathophysiology of heart failure (HF) with preserved ejection fraction (HFpEF) is suggested to be influenced by inflammation. Leukocyte indices, including the neutrophil–lymphocyte ratio (NLR), the monocyte–lymphocyte ratio (MLR), and the pan-immune inflammation value (PIV), can be utilized as biomarkers of systemic inflammation. Their prognostic utility is yet to be fully understood. **Methods:** Between December 2010 and May 2023, patients presenting to a tertiary referral center for HFpEF were included into a prospective registry. The association of the NLR, MLR, and PIV with the composite endpoint of all-cause mortality and HF-related hospitalization was tested utilizing Cox regression analysis. **Results:** In total, 479 patients (median 74.3, interquartile range (IQR): 69.22–78.3 years, 27.8% male) were included. Patients were observed for 43 (IQR: 11–70) months, during which a total of 267 (55.7%) patients met the primary endpoint. In a univariate Cox regression analysis, an above-the-median NLR implied a hazard ratio (HR) of 1.76 (95%-confidence interval (CI): 1.38–2.24, *p* < 0.001), an MLR of 1.46 (95%-CI: 1.14–1.86, *p* = 0.003), and a PIV of 1.67, 95%-CI: 1.30–2.13, *p* < 0.001) for the composite endpoint. After adjustment in a step-wise model, the NLR (HR: 1.81, 95%-CI: 1.22–2.69, *p* = 0.003), the MLR (HR: 1.57, 95%-CI: 1.06–2.34, *p* = 0.026), and the PIV (HR: 1.64, 95%-CI: 1.10–2.46, *p* = 0.015) remained significantly associated with the combined endpoint. **Conclusions:** The NLR, the MLR, and the PIV are simple biomarkers independently associated with outcomes in patients with HFpEF.

## 1. Introduction

In heart failure (HF) with preserved ejection fraction (HFpEF), insufficient cardiac output is attributed to impairment of ventricular relaxation and loss of myocardial compliance [1]. While the mechanisms resulting in diastolic dysfunction and concomitant myocardial remodeling remain to be fully elucidated, some indications suggest that microvascular endothelial dysfunction and systemic inflammation may contribute to the pathogenesis of HFpEF [1].

Systemic inflammation assessed using a panel of advanced biomarkers has been demonstrated to be associated with adverse outcomes and exerts influence on left ventricular relaxation and hemodynamic parameters [2,3]. Therefore, a correlation between biomarkers of inflammation and the degree of diastolic dysfunction, congestion, and, ultimately, outcome may be assumed [2,3,4]. However, the markers utilized in previous studies on inflammation in patients with HFpEF, e.g., lipocalin 2, urokinase receptor, galectin 9, interleukin 8, tumor necrosis factor receptor, insulin-like growth factor binding protein 7, and fibroblast growth factor 23 are not routinely available [2,3,4].

The neutrophil–lymphocyte ratio (NLR), the monocyte–lymphocyte ratio (MLR), and the pan-immune inflammation value (PIV) are alternative biomarkers of inflammation. While the prognostic value of the NLR in HFpEF has been explored previously [5], this association is less clear for the MLR and has not yet been demonstrated for the PIV.

We therefore aimed to evaluate the prognostic implications of three leukocyte indices, i.e., the NLR, the MLR, and the PIV, for all-cause mortality and HF-related hospitalizations in a real-world cohort of HFpEF patients.

## 2. Methods

### 2.1. Setting

This retrospective analysis was conducted within the scope of a prospective registry of patients with HFpEF at the Medical University of Vienna, Department of Internal Medicine II, Division of Cardiology. The study was approved by the local ethics committee (#1079/2023) and was implemented in compliance with the Declaration of Helsinki. Prior to their inclusion, all patients provided written informed consent.

### 2.2. Subjects and Study Design

Patients were recruited between December 2010 and May 2023 at the institution’s outpatient tertiary referral center for HFpEF, the central echocardiography laboratory, and after admission for decompensated acute HFpEF and subsequent decongestion.

For inclusion in the analysis, consecutive patients were screened for eligibility. Patients had to meet the following inclusion criteria: (1) definite diagnosis of HFpEF as described below, (2) ability and willingness to undergo in-clinic assessments and imaging studies, (3) evidence of a differential hemogram within ± one month to inclusion, and (4) evidence of personally signed informed consent. Patients were excluded if (1) a cardiac myopathy other than HFpEF, i.e., cardiac amyloidosis, hypertrophic (obstructive) cardiomyopathy, cardiac sarcoidosis, hemochromatosis, Morbus Fabry, pericardial disease, or valvular heart disease were considered the primary cause of HF.

### 2.3. Diagnostic Criteria

A diagnosis of HFpEF was established in patients who met the diagnostic criteria of the most recent guidelines on HF by the European Society of Cardiology: (1) signs and symptoms of HF, (2) echocardiographic evidence of preserved ejection fraction (≥50%), and (3) structural, functional or serologic findings consistent with ventricular diastolic dysfunction [6]. Detailed thresholds and a description of findings included in the diagnostic criteria are depicted in Supplementary Table S1.

In addition, the H_2_FPEF [7] and HFA-PEFF [8] scores were calculated. Both scores are computed by summing up points from clinical (age, sex, heart rhythm, body mass index), laboratory (N-terminal prohormone of brain-natriuretic peptide (NT-proBNP)), and echocardiographic (pulmonary hypertension, e’ values, global longitudinal strain, wall thickness) assessments and aim to calculate the likelihood of a diagnosis of HFpEF [7,8].

### 2.4. Study Procedures

Baseline evaluations included a clinical evaluation, an extensive laboratory assessment featuring blood chemistry, biomarkers of HF, and an automated differential hemogram. Blood was drawn utilizing ethylenediaminetetraacetic acid-containing blood sampling tubes and transferred to the Department of Laboratory Medicine at the Medical University of Vienna, where the analysis was conducted. For the differential blood count, Sysmex XE and XN series hematology systems (Sysmex Corporation, Kobe, Japan) were utilized. For the estimation of the glomerular filtration rate (eGFR), the Chronic Kidney Disease Epidemiology Collaboration (CKD-EPI) equation was utilized. Baseline evaluations were performed at the initial patient encounter both at the outpatient clinic and in patients diagnosed with or undergoing evaluation or treatment for HFpEF.

Transthoracic echocardiography was performed by board-certified and experienced operators on high-end machines (GE Vivid 95 and Vivid 7; GE Healthcare, Wauwatosa, WI, USA) in accordance with contemporary recommendations [9,10]. Image analysis was performed post hoc on an offline clinical workstation equipped with dedicated software (EchoPAC, Version 206, GE Healthcare, Wauwatosa, WI, USA).

### 2.5. Parameters of Interest

Leukocyte indices were calculated from the laboratory assessment performed at baseline. The NLR was calculated by dividing the absolute number of neutrophils (G/L) by the absolute number of lymphocytes (G/L) in the complete hemogram. The MLR was calculated analogously by dividing the absolute number of monocytes (G/L) by the absolute number of lymphocytes (G/L). The PIV was calculated by multiplying the neutrophil count, the monocyte count, and the platelet count and then dividing by the lymphocyte count (G/L for all).

### 2.6. Endpoints

The primary endpoint for this analysis was a composite endpoint consisting of both all-cause mortality and HF-related hospitalization [5]. In addition, the secondary endpoint of all-cause mortality was explored.

The Austrian national statistics authority’s (Statistics Austria) death registry was queried for the compilation of mortality data. In addition, electronic health records were retrieved if available. Data on HF-related hospitalizations was documented by follow-up in our outpatient clinic. In addition, electronic health records were screened for clinical events. Hospitalizations were adjudicated as HF-related if patients were admitted due to dyspnea, weight gain, or peripheral edema and required intravenous diuretic therapy as described previously.

### 2.7. Statistical Analysis

Categorial variables are presented as numbers and percentages, continuous variables as mean and standard deviation (SD) or median and interquartile range (IQR) depending on the individual distribution, which was assessed utilizing the Kolmogorov–Smirnov test. Characteristics between the outcome groups were compared using the chi-square test and the *t*-test or the Mann–Whitney U-test as applicable. The leukocyte indices’ correlation with clinical parameters, biomarkers of HF, the eGFR, and imaging assessments, as well as the H_2_FPEF [7] and HFA-PEFF [8] scores, were tested using Spearman correlation analysis. Predictors of the leukocyte indices were explored using linear regression analysis. The prognostic utility was assessed using a step-wise Cox proportional hazard regression analysis. First, all parameters were tested in a univariate model. Then, parameters with significant predictive value in the univariate Cox regression were included in a multivariate regression model with the leukocyte indices’ medians.

With all statistical analysis, significance was assumed with confidence intervals (CI) of 95% and *p*-values of <0.05. For data analysis, IBM SPSS Statistics 29 (IBM Corporation, Armonk, NY, USA) & STATA 15.1 (StataCorporation, College Station, TX, USA) were utilized.

## 3. Results

**Figure 1:**
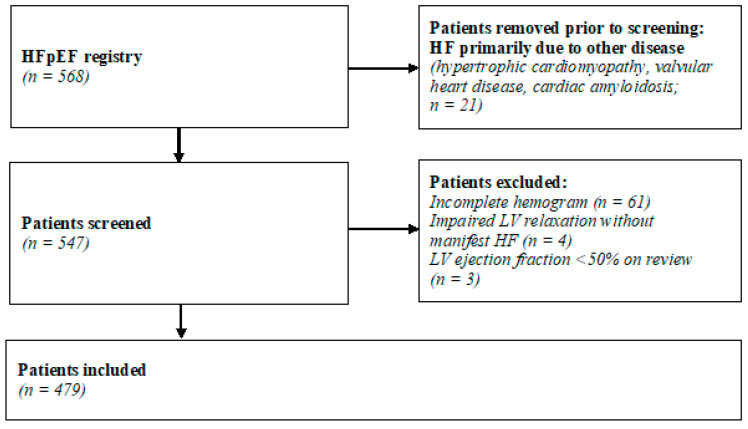
The patient recruitment process. HF indicates heart failure; HFpEF, heart failure with preserved ejection fraction; LV, left ventricular.

After screening 568 patients, the final cohort comprised 479 individuals. The patient recruitment process is illustrated in Figure 1.

### 3.1. Baseline Characteristics

**Figure 2:**
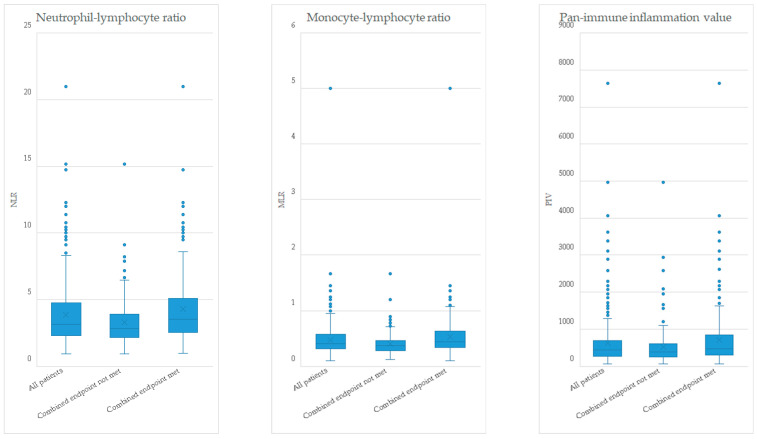
Boxplots—Leukocyte indices at baseline. MLR indicates monocyte–lymphocyte ratio; NLR, neutrophil–lymphocyte ratio; PIV, pan-immune inflammation value.

Patients were 74 (IQR: 69–78) years old, and about a quarter of patients (133, 28%) were male. Overall, disease severity was advanced as 281 (61.5%) patients were in New York Heart Association (NYHA) functional class III. The median level of NT-proBNP was 1079 (IQR: 419–2062), and the median eGFR was 55 mL/min/1.73 m^2^ (IQR: 41–70). The most frequent comorbidities were arterial hypertension, atrial fibrillation, and diabetes mellitus. The median NLR was 3.2 (IQR: 2.3–4.8), the median MLR 0.40 (IQR: 0.31–0.57), and the median PIV 433 (272–696). Detailed patient baseline characteristics are depicted in Table 1 and Figure 2.

### 3.2. Association with Clinical Characteristics and Biomarkers of Heart Failure

Statistically significant correlations with the NLR, the MLR, and the PIV were observed for age, hemoglobin, C-reactive protein, lactate dehydrogenase, and biomarkers of HF and kidney function. In addition, all three indices demonstrated a statistically significant correlation with systolic pulmonary artery pressure. However, correlations were generally weak. The full correlation analysis can be found in the Supplementary Table S2. Supplementary Tables S3–S5 depict a step-wise regression analysis for the NLR, the MLR, and the PIV. C-reactive protein was the only parameter to predict all three indices in multivariate analysis and also the only tested parameter with a statistically significant association with the PIV in multivariate analysis.

### 3.3. Outcome

The patient cohort was observed for 43 (IQR: 11–70) months. During the follow-up period, a total of 267 (55.7%) patients met the combined endpoint of all-cause mortality and HF-related hospitalizations after a median of 23 (IQR: 5–50) months. A total of 197 (41.1%) patients were deceased after 41 (IQR: 20–70) months, and 181 (37.8%) patients were hospitalized for HF after 17 (IQR: 4–40) months.

Association with the combined endpoint was demonstrated for all tested leukocyte indices in univariate analysis. An above-the-median NLR implied a hazard ratio (HR) of 1.76 (95%-CI: 1.38–2.24, *p* < 0.001), an above-median MLR of 1.46 (95%-CI: 1.14–1.86, *p* = 0.003), and a HR of 1.67 (95%-CI: 1.30–2.13, *p* < 0.001) for the above-median PIV.

**Figure 3:**
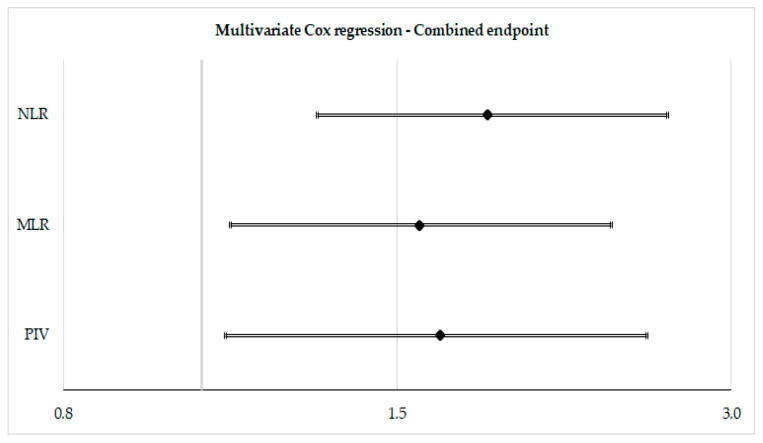
Forest plot—multivariate Cox regression for the combined endpoint.

**Figure 4:**
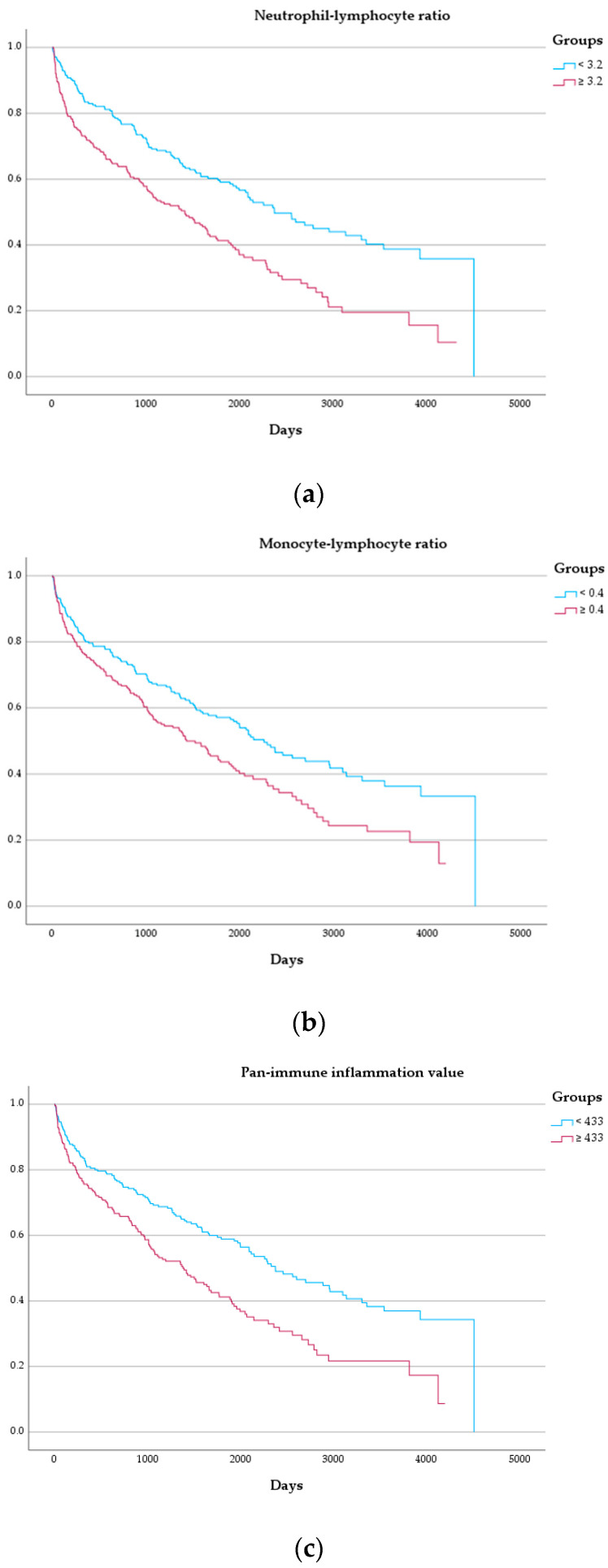
Kaplan–Meier curves for the combined endpoint. (**a**) Neutrophil–lymphocyte ratio (log rank *p* < 0.001). (**b**) Monocyte–lymphocyte ratio (log rank *p* = 0.002). (**c**) Pan-immune inflammation value (log rank *p* < 0.001).

After assessment in the univariate model, leukocyte indices were tested after adjustment parameters which demonstrated significant association in univariate Cox regression (i.e., sex, NYHA functional class, levels of NT-proBNP, the eGFR, C-reactive protein, history of both atrial fibrillation or diabetes mellitus, systolic pulmonary artery pressure, and left ventricular global longitudinal strain). In these respective multivariate models, the NLR (HR: 1.81, 95%-CI: 1.22–2.69, *p* = 0.003), the MLR (HR: 1.57, 95%-CI: 1.06–2.34, *p* = 0.026), and the PIV (HR: 1.64, 95%-CI: 1.10–2.46, *p* = 0.015) were significantly associated with the combined endpoint. Detailed results of the Cox regression analysis can be found in Table 2 and Figure 3. Figure 4 depicts Kaplan–Meier curves for the combined endpoint with the population split per median of the respective leukocyte indices.

With respect to the secondary endpoint of all-cause mortality, significant association after multivariate adjustment could be demonstrated for the NLR (HR: 1.86, 95%-CI: 1.30–2.66, *p* < 0.001) and the PIV (HR: 1.56, 95%-CI: 1.09–2.24, *p* = 0.016). All results of the Cox regression for the secondary endpoint are depicted in Supplementary Table S6.

## 4. Discussion

Our analysis has demonstrated the independent association of the NLR, MLR, and PIV with all-cause mortality and HF-related hospitalizations in patients with HFpEF.

This is the first study to report the prognostic utility of the MLR and the PIV in patients with HFpEF. While confirming previous findings regarding the association of the NLR with mortality and HF-related hospitalizations [5], this is also the first study to directly compare all three leukocyte indices and assess the determinants of the respective leukocyte indices.

An elevated NLR is associated with disorders of metabolism, including diabetes and hypercholesterinemia, and hypertension [11]. The prognostic utility has so far also been demonstrated in acute coronary syndrome [12], peripheral artery disease [13], cardiovascular surgical interventions [14], Coronavirus disease 2019 pneumonia [15], and various chronic inflammatory conditions [16].

In HF irrespective of ejection fraction, NLR was shown to correlate with HF severity, limited functional capacity, and adverse cardiovascular outcomes, including in-hospital mortality, hospital readmission, need for cardiac resynchronization therapy, and long-term all-cause mortality [5,17,18]. The NLR was a significant independent predictive parameter in a post hoc analysis of patients with new-onset or worsening HF from a large-scale observational population study [5].

Clinical implications of an elevated MLR are only beginning to be explored. In the general population, the MLR seems to be associated with all-cause mortality [19]. In cardiovascular disease, an association with atherosclerosis may be assumed [20]. An increased MLR was associated with the burden of disease and HF-related hospitalizations in patients undergoing coronary angiography [21]. Following coronary artery bypass grafting surgery, both the NLR as well as the MLR were associated with new-onset atrial fibrillation [22].

While the PIV is currently utilized mainly in oncology, the value of the PIV for cardiovascular disease is only beginning to be recognized [23]. In acute decompensated patients with heart failure with reduced ejection fraction, the PIV also been demonstrated to provide significant additional prognostic value [24]. A recent study also suggests that in patients following percutaneous coronary intervention for ST-segment elevation myocardial infarction, the PIV may also be utilized as a prognostic marker [25]. As with MLR, there also seems to be an association with the severity of atherosclerosis [26].

### 4.1. Clinical Implications

As a differential blood count is more easily available and more cost-efficient than advanced biomarkers of inflammation in most clinical settings, our findings may support the incorporation of leukocyte indices into future scoring and risk prediction systems in HFpEF and potentially beyond. In clinical practice, an elevated NLR might prompt the assessment of more advanced biomarkers of inflammation and eventually the referral to specialized centers, where patients at increased risk could receive intensified monitoring and therapy. As anti-inflammatory therapy for HFpEF is beginning to be explored [27,28], the utility of leukocyte indices for patient selection or monitoring ought to be assessed.

### 4.2. Pathophysiologic Considerations

It is currently unclear which exact factors determine the tested leukocyte indices and whether an elevation in NLR, MLR, or PIV is to be interpreted as a mere indicator of inflammation or a contributor to the pathogenesis of HFpEF.

In our analysis, C-reactive protein was the only factor consistently associated with an elevation in all leukocyte indices in multivariate regression analysis. However, levels of correlation of leukocyte indices and levels of C-reactive protein were generally weak, indicating that C-reactive protein and the NLR, MLR, and PIV could be affected by different pathways. Beyond the count of leukocytes and individual ratios, leukocyte activation, degranulation, and release of pro-inflammatory mediators, e.g., myeloperoxidase or neutrophil extracellular traps, are considered to contribute to the pathogenesis of HFpEF [29].

Myeloperoxidase is an enzyme linked to the generation of neutrophil-derived reactive oxygen species and is suggested to be a key contributor to the inflammatory aspects of atherogenesis and the formation of abdominal aortic aneurysms [30]. It is currently also under scrutiny as a potential target for the attenuation of inflammation in HFpEF patients [27] as proteomic analysis has uncovered the ability of a myeloperoxidase inhibitor to reduce the activation of pro-inflammatory pathways which are linked to adverse outcomes in HFpEF [31].

Neutrophil extracellular traps, for the release of which, myeloperoxidase is a significant contributor [32], have been demonstrated to contribute to inflammation and endothelial dysfunction [33,34]. Previous studies have identified a high prevalence of coronary microvascular dysfunction in HFpEF patients and linked it to adverse outcomes, myocardial edema, myocardial hypertrophy, diastolic dysfunction, and atrial fibrillation [2,35,36].

In an observational study of HF patients and healthy controls, neutrophils were characterized as high- versus low-density, the latter disproportionally elevated in HF patients compared to high-density neutrophils [37]. In addition, low-density neutrophils also expressed more adhesive neutrophil extracellular traps [37]. Bai et al. demonstrated that in patients with HFpEF, both neutrophil elastase and activation of genes associated with the degranulation of neutrophils were increased compared to control subjects [29].

The nucleotide-binding domain, leucine-rich–containing family, pyrin domain-containing-3 (NLRP3) inflammasome is a key component of the innate immune system activating several pro-inflammatory downstream pathways involved in cardiovascular disease and comorbidities associated with HFpEF including obesity, diabetes mellitus, hypertension, and chronic obstructive pulmonary disease [38]. In HFpEF patients with atrial fibrillation, NLRP3 inflammasome expression levels were significantly elevated compared to patients with atrial fibrillation alone and also correlated with the H2FPEF score [39]. By inhibiting the NLRP3 inflammasome in a murine model, Cheng et al. achieved improved exercise capacity, left ventricular diastolic function, and reduced glucose intolerance, lowering the levels of pro-inflammatory cytokines and attenuating adverse myocardial remodeling [40]. Another study found that elevated levels of β-hydroxybutyrate, a common ketone body, can reduce mitochondrial dysfunction by attenuation of NLRP3 inflammasome activation [41]. With their mild pro-ketogenic properties, sodium-glucose cotransporter 2 inhibitors have been shown to lower the activity of pro-inflammatory pathways, which may constitute an underappreciated aspect of gliflozin therapy in HF [42,43,44].

### 4.3. Future Directions

Further studies will need to establish the pathophysiologic implications of an elevated NLR, MLR, and PIV for inflammation, immuno-composition, and resulting diastolic dysfunction or fibrosis in HFpEF. Special interest may be directed at exploring the properties of different neutrophil sub-populations in the context of HFpEF specifically and inflammation in the context of cardiovascular disease more broadly.

Besides leukocyte indices as markers of inflammation, alternative ratios have been suggested as markers of disease severity in HFpEF, e.g., the albumin-to-gamma-glutamyl-transferase ratio, the white-blood-cell-count-to-mean-platelet-volume ratio, and the high-density-lipoprotein-cholesterol-to-C-reactive-protein ratio, some of which have also been demonstrated to directly correlate with echocardiographic signs of diastolic dysfunction [45].

Advanced imaging studies, including tissue Doppler and strain echocardiography and cardiac magnetic resonance imaging, will be needed to further assess the impact of elevated leukocyte indices indicative of inflammation on cardiac function and myocardial tissue composition. A direct comparison between leukocyte indices and advanced biomarkers of HF could aid the selection of additional parameters to increase the accuracy of diagnostic and prognostic scores in HFpEF.

In a broader context, more detailed knowledge of the pathways involved in the pathogenesis of HFpEF may aid the development of novel therapeutic agents to intercept adverse inflammatory signals and ultimately delay the progression of HF in these patients.

### 4.4. Limitations

Due to the retrospective nature of our analysis, a number of limitations need to be considered. First, as with all retrospective analyses, certain confounding factors unbeknownst to the investigators cannot be fully excluded. Secondly, the analysis was conducted within the scope of a single-center prospective registry. Therefore, the results’ external validity remains to be confirmed in other HFpEF populations. Thirdly, flow cytometry was not performed in our study but may aid the differentiation of sub-populations of neutrophils, monocytes, and lymphocytes which may play different roles in inflammation in the context of HFpEF. Lastly, only a small proportion of patients have received sodium-glucose-cotransporter 2 inhibitors. This may limit the applicability of findings in contemporary HFpEF patients as gliflozins have been suggested to influence systemic inflammation and myocardial remodeling as described previously [46].

## 5. Conclusions

The NLR, the MLR, and the PIV are independently associated with a combined endpoint of all-cause mortality and HF-related hospitalizations as well as all-cause mortality in patients with HFpEF.

## Figures and Tables

**Table 1:** The patient cohort baseline characteristics.

Variable	All Patients (n = 479)	Combined Endpoint Met (n = 267)	Combined Endpoint Not Met (n = 212)	*p*-Value
**Demographics**				
Age, median (IQR)	74.3 (69.2–78.3)	73.9 (68.5–78.6)	74.5 (69.3–78.3)	0.734
Male sex, n (%)	133 (27.8%)	88 (33.0%)	45 (21.2%)	0.004
**Clinical parameters**				
BMI, median (IQR)	29.1 (25.3–33.3)	29.3 (25.7–33.8)	28.8 (25.0–32.0)	0.212
NYHA functional class				<0.001
1	17 (3.7%)	4 (1.6%)	13 (6.3%)	
2	159 (34.8%)	59 (23.5%)	100 (48.5%)	
3	281 (61.5%)	188 (74.9%)	93 (45.2%)	
Heart rate, bpm, median (IQR)	70 (62–80)	72 (62–81)	68 (61–79)	0.155
BP systolic, mmHg, median (IQR)	140 (125–155)	140 (122–156)	140 (125–151)	0.908
BP diastolic, mmHg, median (IQR)	80 (70–87)	78 (69–86)	80 (70–88)	0.036
NT-proBNP, pg/mL, median (IQR)	1068 (415–2062)	1498 (720–2577)	643 (319–1232)	<0.001
Creatinine, mg/dL, median (IQR)	1.09 (0.88–1.37)	1.17 (0.93–1.50)	0.98 (0.84–1.20)	<0.001
eGFR, mL/min/1.73 m^2^, median (IQR)	55 (41–71)	49 (37–66)	62 (46–76)	<0.001
**Heart failure with preserved ejection fraction scores**				
H2FPEF-Score				0.132
≤3	99 (20.7%)	44 (16.5%)	55 (25.9%)	
4–6	231 (48.2%)	134 (50.2%)	97 (45.8%)	
7–9	149 (31.1%)	89 (33.3%)	60 (28.3%)	
HFA-PEFF-Score				<0.001
≤2	55 (11.5%)	21 (7.9%)	34 (16.0%)	
3–4	124 (25.9%)	57 (21.3%)	67 (31.6%)	
5–6	300 (62.6%)	189 (70.8)	111 (52.4%)	
**Comorbidities and medical history**				
Atrial fibrillation, n (%)	289 (60.3%)	170 (63.7%)	119 (56.1%)	0.094
Significant coronary artery disease, n (%)	153 (32.0%)	100 (37.5%)	53 (25.1%)	0.004
Myocardial infarction, n (%)	36 (7.6%)	24 (9.1%)	12 (5.8%)	0.177
Diabetes mellitus, n (%)	164 (34.3%)	114 (42.9%)	50 (23.6%)	<0.001
Arterial hypertension, n (%)	441 (92.3%)	252 (94.4%)	189 (89.6%)	0.051
**Heart failure medication**				
Beta receptor antagonists, n (%)	345 (72.2%)	200 (74.9%)	145 (68.7%)	0.134
ACEi/AT1i, n (%)	319 (66.7%)	176 (65.9%)	143 (67.8%)	0.669
Mineralocorticoid receptor antagonists, n (%)	206 (43.2%)	128 (47.9%)	78 (37.1%)	0.018
Sodium-glucose-cotransporter 2 inhibitors, n (%)	18 (3.8%)	3 (1.1%)	15 (7.1%)	<0.001
**Echocardiographic markers**				
IVS, mm, median (IQR)	12 (11–14)	12 (11–14)	12 (11–14)	0.888
RWT, median (IQR)	0.48 (0.4–0.55)	0.49 (0.41–0.56)	0.47 (0.38–0.55)	0.055
LV ejection fraction, %, median (IQR)	60 (55–66)	60 (55–66)	60 (54–66)	0.990
LA volume index, mL/m^2^, median (IQR)	40 (30–53)	40 (32–55)	38 (29–48)	0.002
LV stroke volume index, mL/m^2^, median (IQR)	24 (19–30)	25 (19–31)	23 (18–29)	0.035
LV mass index, g/m^2^, median (IQR)	94 (78–115)	94 (79–115)	96 (75–114)	0.426
E/A, median (IQR)	1.19 (0.84–2.04)	1.42 (0.92–2.28)	1.07 (0.79–1.55)	0.001
E/e’, median (IQR)	12.1 (9.1–15.1)	13.6 (9.8–15.6)	10.9 (8.6–14.0)	0.086
sysPAP, mmHg, median (IQR)	53 (43–67)	62 (48–74)	46 (37–57)	<0.001
LV global longitudinal strain, -%, mean (SD)	16.6 (3.8)	16.1 (3.6)	17.2 (3.9)	0.019
**Markers of inflammation**				
Leukocytes, G/L, median (IQR)	7.3 (5.9–8.7)	7.3 (5.7–8.9)	7.2 (5.9–8.4)	0.335
Neutrophils, G/L, median (IQR)	4.7 (3.9–6.0)	4.9 (4.0–6.1)	4.5 (3.7–5.8)	0.038
Monocytes, G/L, median (IQR)	0.6 (0.5–0.7)	0.6 (0.5–0.7)	0.6 (0.5–0.7)	0.037
Lymphocytes, G/L, median (IQR)	1.5 (1.1–1.9)	1.4 (1.0–1.8)	1.6 (1.2–2.0)	<0.001
Thrombocytes, G/L, median (IQR)	226 (187–270)	223 (185–269)	228 (195–272)	0.172
C-reactive protein, mg/dL, median (IQR)	0.37 (0.16–0.8)	0.49 (0.21–1.12)	0.28 (0.11–0.57)	<0.001
NLR, median (IQR)	3.2 (2.3–4.8)	3.6 (2.5–5.1)	2.8 (2.2–3.9)	<0.001
MLR, median (IQR)	0.40 (0.31–0.57)	0.43 (0.33–0.64)	0.38 (0.29–0.46)	<0.001
PIV, median (IQR)	433 (272–696)	469 (307–840)	385 (249–605)	<0.001

ACEi indicates angiotensin-converting enzyme inhibitor; AT1i, angiotensin receptor 1 inhibitor; BMI, body mass index; BP, blood pressure; bpm, beats per minute; E/A, E-wave-A-wave ratio; E/e’, E-wave-E-prime ratio; eGFR, estimated glomerular filtration rate; IQR, interquartile range; IVS, interventricular septum; LA, left atrium; LV, left ventricle; MLR, monocyte–lymphocyte ratio; mmHg, millimeters of mercury; NLR, neutrophil–lymphocyte ratio; NT-proBNP, N-terminal prohormone of brain natriuretic peptide; NYHA, New York heart association staging system; PIV, pan-immune inflammation value; RWT, relative wall thickness; SD, standard deviation; sysPAP, systolic pulmonary artery pressure; For normally distributed continuous variables, mean and standard deviation are reported, and the *t*-test was used for comparison; for non-normally distributed variables, median and standard deviation are reported and the Mann–Whitney U-test was utilized.

**Table 2:** Cox regression for the combined endpoint of all-cause mortality and heart failure-related hospitalizations.

Variable	HR	95%-CI	*p*-Value	HR	95%-CI	*p*-Value	HR	95%-CI	*p*-Value	HR	95%-CI	*p*-Value
	Univariate			Multivariate NLR			Multivariate MLR			Multivariate PIV		
NLR median	1.76	1.38–2.24	<0.001	1.81	1.22–2.69	0.003						
MLR median	1.46	1.14–1.86	0.003				1.57	1.06–2.34	0.026			
PIV median	1.67	1.30–2.13	<0.001							1.64	1.10–2.46	0.015
Age	1.01	1.00–1.03	0.093									
Male sex	1.50	1.12–1.88	0.004	1.98	1.31–3.00	0.001	1.91	1.26–2.89	0.002	2.01	1.32–3.05	0.001
BMI	1.02	1.00–1.04	0.137									
NYHA	2.36	1.87–2.98	<0.001	1.83	1.31–2.57	<0.001	1.86	1.34–2.60	<0.001	1.80	1.28–2.54	<0.001
NT-proBNP	1.00	1.00–1.00	<0.001	1.00	1.00–1.00	0.233	1.00	1.00–1.00	0.410	1.00	1.00–1.00	0.240
eGFR	0.98	0.98–0.99	<0.001	0.99	0.98–1.00	0.005	0.99	0.98–1.00	0.004	0.99	0.98–1.0	0.005
CRP	1.08	1.03–1.14	0.001	1.01	0.91–1.12	0.852	1.02	0.92–1.13	0.731	1.01	0.90–1.12	0.930
AF	1.32	1.03–1.70	0.029	0.63	0.42–0.96	0.029	0.68	0.45–1.02	0.064	0.68	0.46–1.02	0.063
DM	1.92	1.51–2.46	<0.001	1.82	1.24–2.69	0.002	1.92	1.30–2.83	0.001	1.92	1.30–2.83	0.001
sysPAP	1.03	1.02–1.04	<0.001	1.03	1.02–1.04	<0.001	1.03	1.02–1.04	<0.001	1.03	1.02–1.04	<0.001
LV GLS	1.05	1.01–1.10	0.022	1.00	0.95–1.05	0.976	1.01	0.96–1.06	0.803	1.00	0.95–1.05	0.970

AF indicates atrial fibrillation; BMI, body mass index; CI, confidence interval; CRP, C-reactive protein; DM, diabetes mellitus; eGFR, estimated glomerular filtration rate; HR, hazard ratio; LV GLS, left ventricular global longitudinal strain; MLR, monocyte–lymphocyte ratio; NLR, neutrophil–lymphocyte ratio; NT-proBNP, N-terminal prohormone of brain natriuretic peptide; NYHA, New York Heart Association stage; PIV, pan-immune inflammation value; sysPAP, systolic pulmonary artery pressure.

## Data Availability

The data supporting this research may be shared upon reasonable request to the corresponding author.

## Supplementary Materials

The following supporting information can be downloaded at: https://www.mdpi.com/article/10.3390/jcm13195875/s1, Table S1. Objective evidence of cardiac structural, functional, and serological abnormalities consistent with the presence of left ventricular diastolic dysfunction/raised left ventricular filling pressures, adapted from McDonagh et al. [6]; Table S2. Correlation analysis of leukocyte indices with patient characteristics; Table S3. Linear regression analysis demonstrating the association between patient characteristics and the neutrophil–lymphocyte ratio; Table S4. Linear regression analysis demonstrating the association between patient characteristics and the monocyte-lymphocyte ratio; Table S5. Linear regression analysis demonstrating the association between patient characteristics and the pan-immune inflammation value; Table S6. Cox regression model for all-cause mortality.
