# Supplementary material for: Leukocyte Indices as Markers of Inflammation and Predictors of Outcome in Heart Failure with Preserved Ejection Fraction
Source: J Clin Med. 2024 Oct 2;13(19):5875. doi: 10.3390/jcm13195875 (PMC11477419; doi:10.3390/jcm13195875)
Supplement: Supplementary file 1 [file jcm-13-05875-s001.zip › jcm-3174010-supplementary.pdf]

Supplementary Table S1. Objective evidence of cardiac structural, functional, and serological abnormalities consistent with the presence of left ventricular diastolic dysfunction/raised left ventricular filling pressures, adapted from McDonagh et al. [6].

**Parameter**	**Threshold**
Left ventricular mass index	Female: ≥95 g/m^2
Relative wall thickness	Male: ≥115 g/m^2
	>0.42
Left atrial volume index	Sinus rhythm: >34 mL/m^2
	Atrial fibrillation: >40 mL/m^2
E/e′ ratio at rest	>9
NT-proBNP	Sinus rhythm: >125 pg/mL
	Atrial fibrillation: >365 pg/mL
Systolic pulmonary artery pressure	>35 mmHg
Tricuspid regurgitation velocity	>2.8 m/s

mmHg indicates millimeters of NT-proBNP; N-terminal prohormone of brain natriuretic peptide,

Supplementary Table S2. Correlation analysis of leukocyte indices with patient characteristics.

Variable	Coeff.	*P-Value*	Coeff.	*P-Value*	Coeff.	*P-Value*
	**NLR**		**MLR**		**PIV**	
**Demographics**						
Age	0.177	***<0.001***	0.223	***<0.001***	0.128	***0.005***
Male sex	0.037	*0.422*	0.128	***0.005***	-0.001	*0.984*
BMI	0.027	*0.553*	-0.018	*0.700*	0.055	*0.227*
**Laboratory assessment**						
Hb	-0.211	***<0.001***	-0.228	***<0.001***	-0.192	***<0.001***
Leukocytes	0.272	***<0.001***	0.083	*0.070*	0.560	***<0.001***
Neutrophils	0.584	***<0.001***	0.285	***<0.001***	0.740	***<0.001***
Monocytes	0.115	***0.012***	0.494	***<0.001***	0.622	***<0.001***
Lymphocytes	-0.729	***<0.001***	-0.666	***<0.001***	-0.293	***<0.001***
Thrombocytes	0.045	*0.329*	-0.007	*0.885*	0.488	***<0.001***
CRP	0.211	***<0.001***	0.250	***<0.001***	0.307	***<0.001***
LDH	0.141	***0.003***	0.106	***0.024***	0.134	***0.004***
NT-proBNP	0.273	***<0.001***	0.312	***<0.001***	0.230	***<0.001***
eGFR	-0.212	***<0.001***	-0.198	***<0.001***	-0.176	***<0.001***
**Echocardiography**						
IVS	0.096	*0.040*	0.071	*0.131*	0.031	*0.511*
RWT	0.007	*0.891*	0.018	*0.728*	0.005	*0.929*
LVEF	0.063	*0.183*	0.075	*0.111*	0.064	*0.176*
LAVi	0.092	*0.055*	0.138	***0.004***	0.043	*0.376*
SVi	-0.003	*0.951*	0.022	*0.646*	0.038	*0.435*
LV mass index	-0.016	*0.770*	-0.035	*0.517*	-0.047	*0.380*
E/A	0.083	*0.192*	0.125	*0.051*	0.073	*0.255*
E/e′	0.260	***0.041***	0.187	*0.146*	0.364	***0.004***
LV-GLS	0.118	*0.051*	0.107	*0.079*	0.140	***0.021***
sysPAP	0.212	***<0.001***	0.270	***<0.001***	0.164	***0.002***
**Diagnostic scores**						
H_2FPEF	0.130	***0.004***	0.122	***0.008***	0.059	*0.196*
HFA-PEFF	0.089	*0.053*	0.154	***<0.001***	0.073	*0.113*

BMI indicates body mass index; Coeff., Spearman correlation coefficient; CRP, C-reactive protein; E/A, E-wave-A-wave ratio; E/e′, E-wave-E-prime ratio; eGFR, estimated glomerular filtration rate; GLS, global longitudinal strain; Hb, hemoglobin; IVS, interventricular septum; LAVi, left atrial volume index; LDH, lactate dehydrogenase; LV, left ventricular; LVEF, left ventricular ejection fraction; MLR, monocyte-lymphocyte ratio; NLR; neutrophil—lymphocyte ratio; NT-proBNP, N-terminal prohormone of brain natriuretic peptide; PIV, pan-immune inflammation value; RWT, relative wall thickness; SVi, stroke volume index; sysPAP, systolic pulmonary artery pressure.

Supplementary Table S3. Linear regression analysis demonstrating the association between patient characteristics and the neutrophil–lymphocyte ratio.

Variable	Coeff.	95%-CI	*P-Value*	Coeff.	95%-CI	*P-Value*
	**Univariate**			**Adjusted**		
Age	0.041	0.015 – 0.066	***0.002***	0.037	0.004 – 0.069	***0.028***
Male sex	0.396	-0.072 – 0.863	*0.097*			
BMI	-0.013	-0.047 – 0.021	*0.447*			
NYHA	0.586	0.252 – 0.919	***<0.001***	0.071	-0-324 – 0.466	*0.723*
NT-proBNP	0.000	0.000 – 0.000	***<0.001***	0.000	0.000 – 0.000	*0.117*
eGFR	-0.018	-0.028 – -0.008	***<0.001***	-0.009	-0.023 - -0.004	*0.178*
CRP	0.355	0.234 – 0.476	***<0.001***	0.420	0.235 – 0.605	***<0.001***
AF	0.744	0.321 – 1.168	***<0.001***	0.552	0.040 – 1.064	***0.035***
DM	0.542	0.101 – 0.982	***0.016***	0.778	0.263 – 1.294	***0.003***
sysPAP	0.026	0.013 – 0.039	***<0.001***	0.013	-0.001 – 0.026	*0.072*
LV-GLS	0.052	-0.022 – 0.126	*0.167*			

AF indicates atrial fibrillation; BMI, body mass index; CI, confidence interval; Coeff., coefficient; CRP, C-reactive protein; DM, diabetes mellitus; eGFR, estimated glomerular filtration rate; LV-GLS, left ventricular global longitudinal strain; NT-proBNP, N-terminal prohormone of brain natriuretic peptide; NYHA, New York Heart Association stage; sysPAP, systolic pulmonary artery pressure.

Supplementary Table S4. Linear regression analysis demonstrating the association between patient characteristics and the monocyte-lymphocyte ratio.

Variable	Coeff.	95%-CI	*P-Value*	Coeff.	95%-CI	*P-Value*
	**Univariate**			**Adjusted**		
Age	0.006	0.002 – 0.009	***<0.001***	0.005	0.000 – 0.009	***0.043***
Male sex	0.100	0.039 – 0.162	***0.002***	0.130	0.054 – 0.207	***<0.001***
BMI	-0.002	-0.007 – 0.002	*0.325*			
NYHA	0.066	0.021 – 0.110	***0.004***	0.033	-0.023 – 0.088	*0.247*
NT-proBNP	0.000	0.000 – 0.000	***0.003***	0.000	0.000 – 0.000	*0.496*
eGFR	-0.002	-0.003 – -0.001	***0.005***	-0.002	-0.004 – 0.000	*0.070*
CRP	0.044	0.028 – 0.061	***<0.001***	0.047	0.021 – 0.073	***<0.001***
AF	0.089	0.032 – 0.146	***0.002***	0.055	-0.017 – 0.127	*0.136*
DM	0.040	-0.019 – 0.099	*0.185*			
sysPAP	0.003	0.001 – 0.004	***0.005***	0.001	-0.001 – 0.003	*0.290*
LV-GLS	0.004	-0.007 – 0.016	*0.450*			

AF indicates atrial fibrillation; BMI, body mass index; CI, confidence interval; Coeff., coefficient; CRP, C-reactive protein; DM, diabetes mellitus; eGFR, estimated glomerular filtration rate; LV-GLS, left ventricular global longitudinal strain; NT-proBNP, N-terminal prohormone of brain natriuretic peptide; NYHA, New York Heart Association stage; sysPAP, systolic pulmonary artery pressure.

Supplementary Table S5. Linear regression analysis demonstrating the association between patient characteristics and the pan-immune inflammation value.

Variable	Coeff.	95%-CI	*P-Value*	Coeff.	95%-CI	*P-Value*
	**Univariate**			**Adjusted**		
Age	7.3	0.166 – 15	***0.045***	6.0	-1.4 – 5.9	*0.226*
Male sex	27	-104 – 158	*0.685*			
BMI	-3.8	-13 – 5.6	*0.424*			
NYHA	163	71 – 254	***<0.001***	85	-22 – 191	*0.118*
NT-proBNP	0.026	0.005 – 0.047	***0.016***	0.011	-0.014 – 0.036	*0.383*
eGFR	-2.9	-5.8 – 0.039	*0.053*			
CRP	109	76 – 143	***<0.001***	151	101 – 200	***<0.001***
AF	60	-60 – 179	*0.328*			
DM	77	-46 – 200	*0.221*			
sysPAP	5.1	1.5 – 8.7	***0.006***	2.2	-1.4 – 5.9	*0.226*
LV-GLS	12	-8.6 – 33	*0.252*			

AF indicates atrial fibrillation; BMI, body mass index; CI, confidence interval; Coeff., coefficient; CRP, C-reactive protein; DM, diabetes mellitus; eGFR, estimated glomerular filtration rate; LV-GLS, left ventricular global longitudinal strain; NT-proBNP, N-terminal prohormone of brain natriuretic peptide; NYHA, New York Heart Association stage; sysPAP, systolic pulmonary artery pressure.

Supplementary Table S6. Cox regression model for all-cause mortality.

Variable	HR	95%-CI	*P-Value*	HR	95%-CI	*P-Value*	HR	95%-CI	*P-Value*	HR	95%-CI	*P-Value*
	**Univariate**			**Multivariate NLR**			**Multivariate MLR**			**Multivariate PIV**		
**NLR median**	2.12	1.58 – 2.82	***<0.001***	1.86	1.30 – 2.66	***<0.001***						
**MLR median**	1.62	1.22 – 2.15	***<0.001***				1.23	0.86 – 1.75	*0.266*			
**PIV median**	1.90	1.43 – 2.54	***<0.001***							1.56	1.09 – 2.24	***0.016***
Age	1.02	1.00 – 1.04	*0.057*									
Male sex	1.48	1.10 – 1.98	***0.009***	1.75	1.21 – 2.52	***0.003***	1.71	1.19 – 2.46	***0.004***	1.78	1.24 – 2.56	***0.002***
BMI	1.00	0.98 – 1.03	*0.701*									
NYHA	2.07	1.62 – 2.66	***<0.001***	1.72	1.27 – 2.33	***<0.001***	1.71	1.26 – 2.32	***<0.001***	1.68	1.24 – 2.28	***<0.001***
NT-proBNP	1.00	1.00 – 1.00	***<0.001***	1.00	1.00 – 1.00	***0.034***	1.00	1.00 – 1.00	***0.031***	1.00	1.00 – 1.00	***0.014***
eGFR	0.98	0.98 – 0.99	***<0.001***	0.99	0.98 – 1.00	*0.112*	0.99	0.98 – 1.00	*0.068*	0.99	0.98 – 1.00	*0.093*
CRP	1.11	1.05 – 1.18	***<0.001***	1.03	0.93 – 1.15	*0.567*	1.06	0.95 – 1.17	*0.302*	1.03	0.92 – 1.15	*0.600*
AF	1.17	0.87 – 1.56	*0.295*									
DM	1.57	1.18 – 2.09	***0.002***	1.39	0.99 – 1.95	*0.056*	1.145	1.03 – 2.03	***0.032***	1.42	1.01 – 1.99	***0.043***
sysPAP	1.03	1.02 – 1.04	***<0.001***	1.03	1.02 – 1.04	***<0.001***	1.03	1.02 – 1.04	***<0.001***	1.03	1.02 – 1.04	***<0.001***
LV-GLS	1.04	0.99 – 1.09	*0.139*									

AF indicates atrial fibrillation; BMI, body mass index; CI, confidence interval; CRP, C-reactive protein; DM, diabetes mellitus; eGFR, estimated glomerular filtration rate; HR, hazard ratio; LV-GLS, left ventricular global longitudinal strain; MLR, monocyte-lymphocyte ratio; NLR, neutrophil-lymphocyte ratio; NT-proBNP, N-terminal prohormone of brain natriuretic peptide; NYHA, New York Heart Association stage; PIV, pan-immune inflammation value; sysPAP, systolic pulmonary artery pressure.
